# Identification for antitumor effects of tramadol in a xenograft mouse model using orthotopic breast cancer cells

**DOI:** 10.1038/s41598-021-01701-9

**Published:** 2021-11-11

**Authors:** Myoung Hwa Kim, Jeong-Rim Lee, Ki-Joon Kim, Ji Hae Jun, Hye Jeong Hwang, Wootaek Lee, Seung Hyun Nam, Ju Eun Oh, Young Chul Yoo

**Affiliations:** 1 Department of Anesthesiology and Pain Medicine, Anesthesia and Pain Research Institute, Yonsei University College of Medicine, Gangnam Severance Hospital, Gangnam-gu, Seoul, 06273 Republic of Korea; 2 Department of Anesthesiology and Pain Medicine, Severance Hospital, Anesthesia and Pain Research Institute, Yonsei University College of Medicine, 50-1 Yonsei-ro, Seodaemun-gu, Seoul, 03722 Republic of Korea; 3 Anesthesia and Pain Research Institute, Yonsei University College of Medicine, 50-1 Yonsei-ro, Seodaemun-gu, Seoul, 03722 Republic of Korea

**Keywords:** Cancer, Oncology

## Abstract

In our previous research showed that tramadol having potential anti-tumor effect was associated with enhancement of oncological prognosis in patients with breast cancer surgery. As these effects have not been confirmed by clinical dose-regulated animal or prospective human studies, we investigated the anti-tumor effect of tramadol in vivo. Female nude mice orthotopically inoculated with luciferase-expressing MCF-7 cells, were randomly divided into the control (saline), tramadol group 1 (1.5 mg kg^−1^ day^−1^), tramadol group 2 (3 mg kg^−1^ day^−1^), and morphine (0.5 mg kg^−1^ day^−1^) (n = 5/group). Bioluminescence signals after D-luciferin injection, tumor size, and tumor weight were compared among groups after 4 weeks. Estrogen receptor (ER), progesterone receptor (PR), and transient receptor potential vanilloid (TRPV)-1 expression, natural killer (NK) cell activity, and serum interleukin (IL)-1β, tumor necrosis factor (TNF)-α, interferon (IFN)-γ, and interleukin (IL)-6 were then examined. Tumour growth was attenuated in tramadol-treated groups (P < 0.05). NK cell activity was significantly decreased only in the morphine treated group not in sham, control, and tramadol groups. The expression levels of ERα, PRα and β, and TRPV1 were decreased in tramadol group 2 compared with those in the morphine group, but not compared to the control group. Serum levels of IL-6 and TNFα were reduced in both tramadol-treated group 1 and 2 compared to the control group. Overall, clinical dose of tramadol has anti-tumour effects on MCF-7 cell-derived breast cancer in a xenograft mouse model*.*

## Introduction

Breast cancer is one of the most commonly occurring malignant solid tumors and is regarded as the principal cause of cancer-related mortality in women worldwide^1^. Several factors may be associated with breast cancer recurrence or metastasis, including the surgical procedure itself, stress-related hormones, immunosuppression, perioperative pain, and opioid analgesics^2,3^. In particular, most available analgesics can affect tumor progression and the immune system either directly by disrupting cellular mechanisms, or indirectly by interacting with the endocrine or sympathetic systems^4,5^. Accordingly, a multimodal analgesic strategy combined with nonopioid agents is the pivotal method for reducing pain in patients after cancer surgery^6^. Moreover, a high dose of opioids may be associated with perioperative immunosuppression and cancer progression^7^.

Tramadol is commonly used to subside pain after surgery. Although the complete mechanism of action of tramadol remains unclear, activation of the μ-opioid receptor and inhibition of the reuptake of monoamines released from nerve endings have been identified as possible mechanisms^8–10^. Our previous retrospective clinical data analysis suggested that tramadol is associated with reduced cancer recurrence and mortality in patients who have undergone surgery for breast cancer^11^. In addition, our in vitro results indicated that tramadol may have a potential anti-tumor effect via several mechanisms that include inhibition of cell proliferation, induction of apoptosis, and regulation of 5-hydroxy tryptamine (HT) receptors and transient receptor potential vanilloid (TRPV)-1 expression^11^. Therefore, we consider that using tramadol as a rescue analgesic could contribute positively to the oncological outcomes in patients with breast cancer.

However, as there have been no animal studies with the clinical dosage of tramadol or prospective human studies, we conducted an in vivo experiment in a mouse xenograft model to confirm the anti-tumor effect and mechanisms of tramadol at the clinical dosage.

## Results

### Measurement of tumor growth in xenograft mice bearing orthotopic MCF-7 tumors

**Figure 1:**
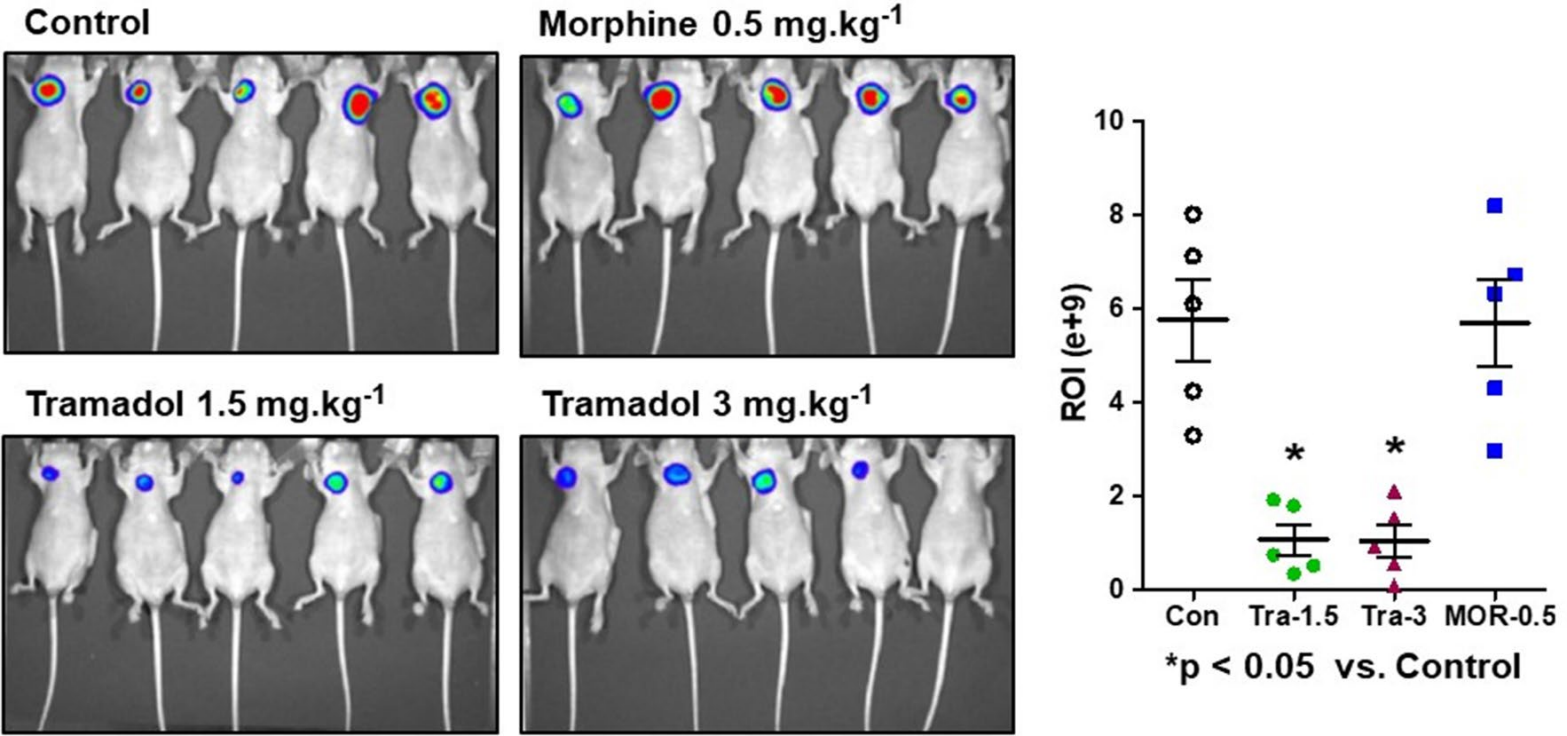
Comparison of tumor growth using the Spectrum in vivo imaging system. Xenografted mice were randomly divided into four different groups with 5 mice per group: control group (Con, saline only), tramadol group 1 (Tra-1.5, 1.5 mg kg^−1^ day^−1^), tramadol group 2 (Tra-3, 3 mg kg^−1^ day^−1^), and morphine group (MOR-0.5, 0.5 mg kg^−1^ day^−1^). Mice bearing MCF7-Luc tumor were intraperitoneally injected with D-luciferin (150 mg kg^−1^). The in vivo imaging results for MCF7-Luc cells are shown for all groups described along with tumor burden. *p < 0.05 *vs*. Con. MCF, Michigan cancer foundation; ROI, Regions of interest.

**Figure 2:**
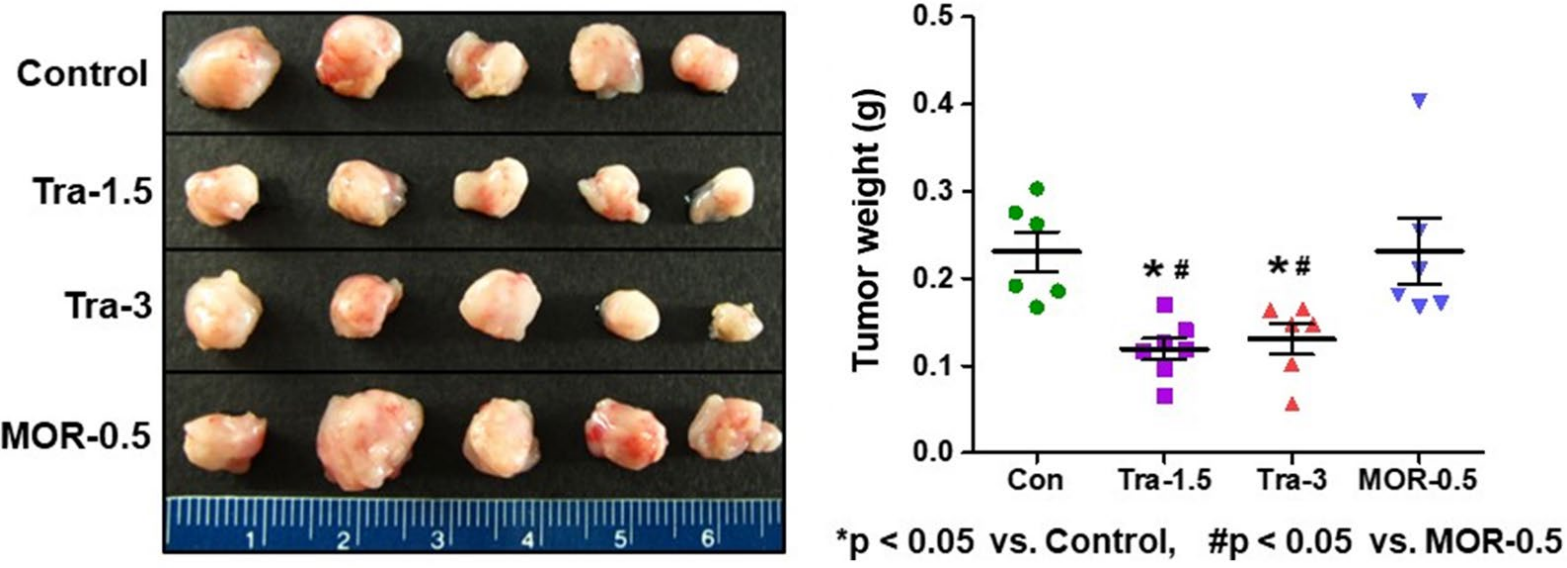
Comparison of tumor size and weight. Tumors were resected from mice 4 weeks after orthotopic inoculation of MCF7-Luc cells. *p < 0.05 *vs*. Con., ^#^p < 0.05 *vs*. MOR-0.5. Con., control group; Tra-1.5, tramadol 1.5 mg kg^−1^ day^−1^; Tra-3, tramadol 3 mg kg^−1^ day^−1^; MOR-0.5, morphine 0.5 mg kg^−1^ day^−1^.

The tumor growth examined using the IVIS in tramadol groups 1 and 2 was significantly inhibited compared with that in the control group (Fig. 1, p < 0.05). Furthermore, the tumor sizes measured using a ruler were smaller in both tramadol groups compared with those in the control and morphine groups. Tumor weights in tramadol groups 1 and 2 were also significantly lower than those in the control and morphine groups (Fig. 2, p < 0.05, repectively). However, no dose-dependent effect was observed between the two tramadol groups.

### Western blot analysis of tumor tissues in xenograft mice bearing orthotopic MCF-7 tumors

**Figure 3:**
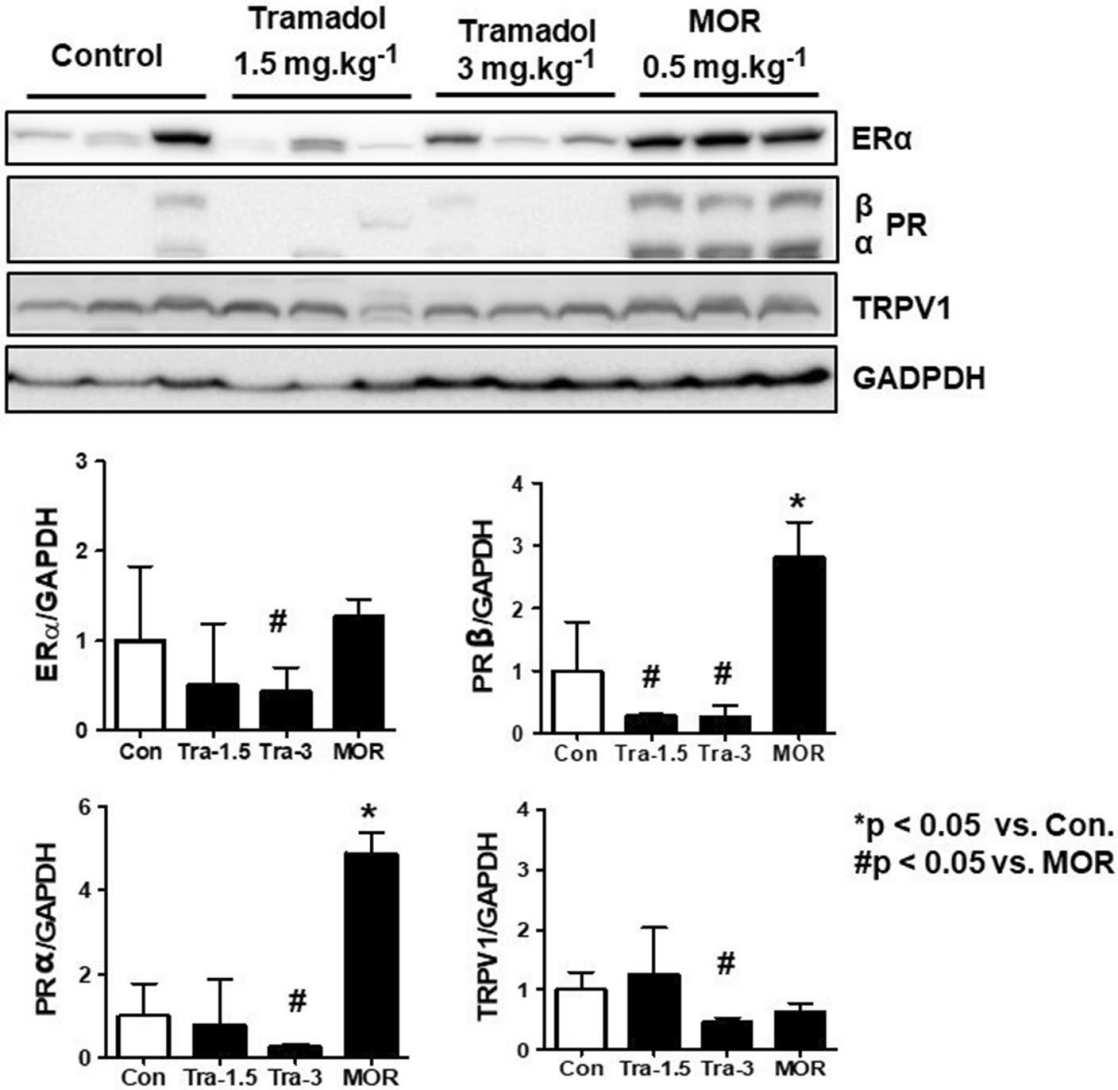
Western blot analysis of MCF-7 tumor tissues from nude mice. Tumors were resected from mice 4 weeks after orthotopic inoculation of MCF7-Luc cells. Representative western immunoblot data for ERα, PR (α or β), TRPV1, and GAPDH. GAPDH were used as the protein loading control. The density of the band was measured using Image J software. *p < 0.05 *vs*. Con., ^#^p < 0.05 vs. MOR-0.5. Con., control group; Tra-1.5, tramadol 1.5 mg kg^−1^ day^−1^; Tra-3, tramadol 3 mg kg^−1^ day^−1^; MOR-0.5, morphine 0.5 mg kg^−1^ day^−1^; MCF, Michigan cancer foundation; ER, estrogen receptor; PR, progesterone receptor; TRPV1, transient receptor potential vanilloid-1; GAPDH, glyceraldehyde-3-phosphate dehydrogenase.

Figure 3 shows the results of western blot analysis for ERα, PRα and β, and TRPV 1 in tumor tissues from mice bearing orthotopic MCF-7 xenografts. The expression levels of ERα, PRα and β, and TRPV1 were decreased in tramadol group 2 compared with those in the morphine group (p < 0.05, respectively). The expression of PRα was also decreased in the tramadol group 1 compared with that in the morphine group (p < 0.05). Moreover, the expression levels of PR α and β were significantly increased in the morphine group compared with those in the control group (p < 0.05). All blots with membrane edges visible as possible and for all replicates were performed in the Supplementary Information file 1 due to lack of images of adequate length of gels/blots in Fig. 3.

### NK cell activity in the plasma of xenograft mice bearing orthotopic MCF-7 tumors

**Figure 4:**
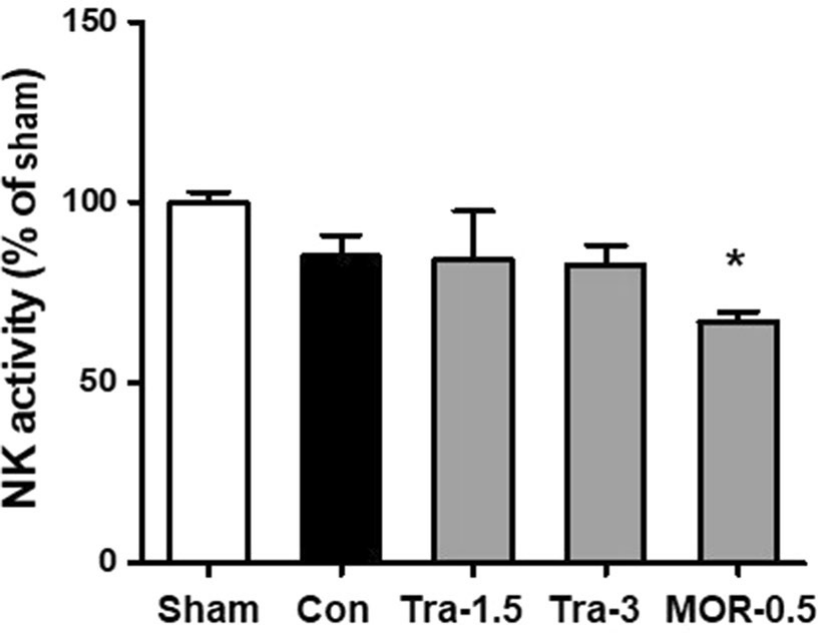
Natural killer cell activity in whole blood of MCF-7 tumor-bearing nude mice. Whole blood samples were collected from mice 4 weeks after orthotopic inoculation of MCF7-Luc cells. Sham, healthy mice not bearing cancer; control group; Tra-1.5, tramadol 1.5 mg kg^−1^ day^−1^; Tra-3, tramadol 3 mg kg^−1^ day^−1^; MOR-0.5, morphine 0.5 mg kg^−1^ day^−1^ *p < 0.05 *vs*. Sham. MCF, Michigan cancer foundation; NK, natural killer.

Figure 4 presents the NK cell activity in the plasma of xenograft mice bearing orthotopic MCF-7 tumors. NK cell activity was comparable between the control and tramadol groups. However, NK cell activity in the morphine group was significantly decreased compared with that in the sham group (100% *vs*. 76.7%, p < 0.05).

### Serum proinflammatory cytokine expression in mice

**Figure 5:**
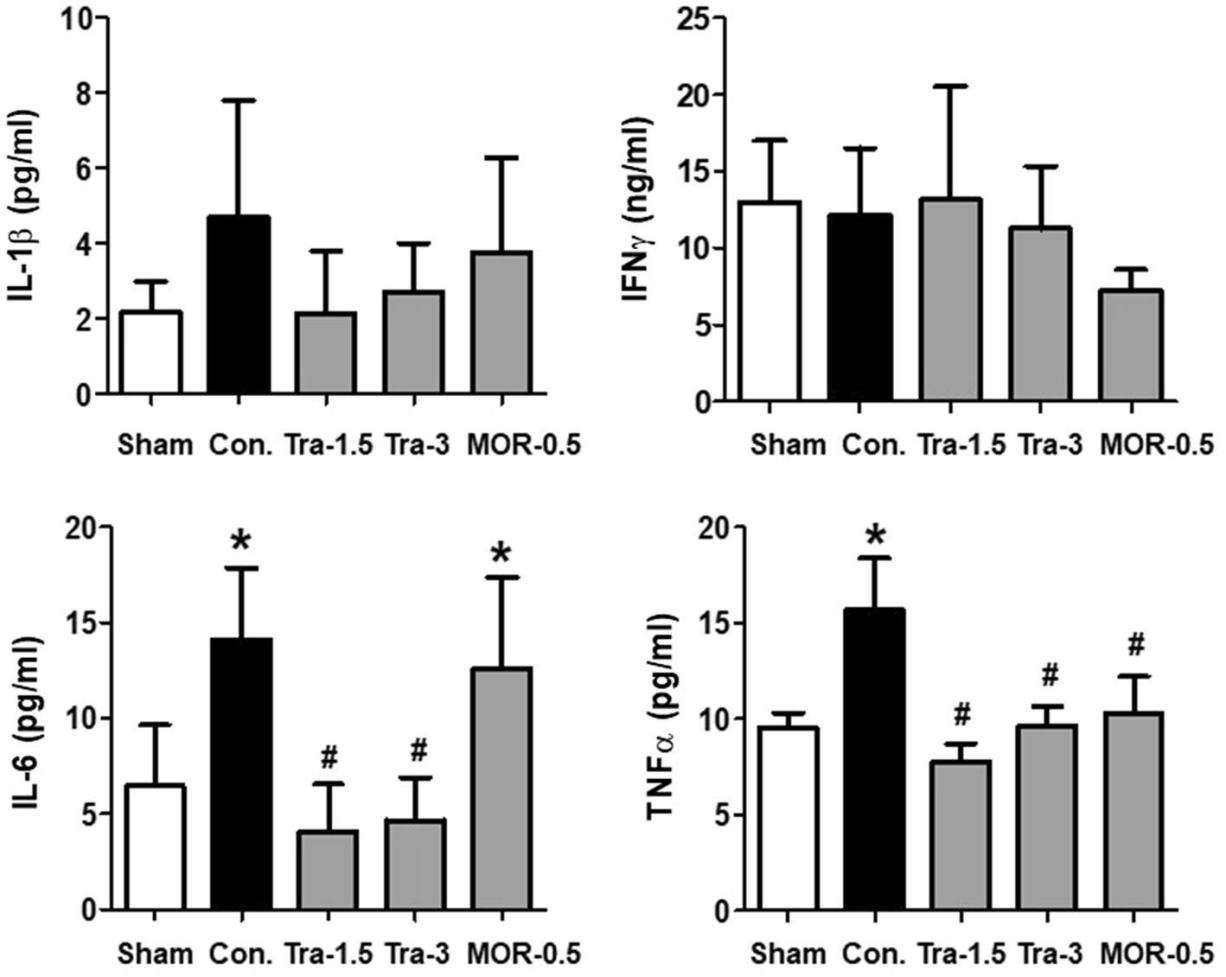
Serum ELISA for MCF-7 tumor-bearing nude mice. Nude mouse serum levels of TNF-α, IFN-γ, IL-6, and IL-1β were measured by ELISA. Sham, healthy mice not bearing cancer; control group; Tra-1.5, tramadol 1.5 mg kg^−1^ day^−1^; Tra-3, tramadol 3 mg kg^−1^ day^−1^; MOR-0.5, morphine 0.5 mg kg^−1^ day^−1^. *p < 0.05 *vs*. Sham, ^#^p < 0.05 vs. Con. MCF, Michigan cancer foundation; TNF; tumor necrosis factor, IFN; interferon, IL; interleukin, ELISA; enzyme-linked immunosorbent assay.

Figure 5 shows the serum levels of TNF-α, INF-γ, IL-6, and IL-1β in xenograft nude mice bearing orthotopic MCF-7 tumors. Among these proinflammatory cytokines, TNF-α and IL-6 levels showed significant differences among the groups. In both the tramadol and morphine groups, the TNF-α serum level was decreased compared with that in the saline-injected (control) group (p < 0.05). Further, the IL-6 serum level was significantly decreased in the tramadol groups 1 and 2 compared with that in the saline-injected (control) group (p < 0.05, respectively). In the control and morphine group, IL-6 levels were significantly higher than those in the sham group (p < 0.05, respectively).

## Discussion

Our previous retrospective study^11^ suggested that tramadol, as a rescue analgesic after breast cancer surgery, was associated with reduced risk of postoperative recurrence and improved survival. Furthermore, the results of an in vitro experiment^11^ demonstrated the anti-tumor effects of tramadol, which could be explained by complex mechanisms including suppression of colony formation, cell cycle arrest, induction of apoptosis, regulation of ER and PR expression, and potential antagonist-like actions on 5-hydroxy tryptamine (HT)_2B_ receptor and TRPV1. This was the first report to demonstrate the impact of tramadol on postoperative oncological outcomes after breast cancer surgery and to identify the mechanism of its anti-tumour activity in breast cancer cells. However, further in vivo studies were needed to precisely confirm how the mechanism identified in vitro plays out in the actual tumor and host microenvironment, and to determine whether the clinical dosage of tramadol is sufficient to obtain a significant effect on the oncological outcomes in vivo in a breast cancer surgery model.

In the present in vivo experiment, we found that the clinical dosage of tramadol could inhibit tumor growth compared to the control (saline) or morphine groups, by examining bioluminescence using IVIS as well as the tumor size and weight. Bioluminescence measurement has been reported as a powerful non-invasive tool for the longitudinal assessment of tumor load in small laboratory animals such as mice^12^. When tumors are close to the surface or growing as a well-defined focal lesion, a strong correlation is observed between the IVIS signal and the actual tumor mass. We observed a significant decrease in the IVIS bioluminescence signal, which corresponded with the direct measurements of tumor size and weight after resection, thereby validating that tramadol inhibited the growth of MCF-7 tumors.

Most breast cancers express ER and/or PR, and their presence in a tumor is well recognized as a favorable prognostic biomarker^13^. Development of drugs targeting these hormone receptors has significantly enhanced the survival of women with hormone receptor-positive breast cancers. As information about ER and PR is vital for patient management, quality assurance is essential to ensure accurate testing^14,15^. Accordingly, we confirmed the downregulatory action of tramadol on ER and PR expression in MCF-7 tumors using both in vitro^11^, and in vivo experiments. TRPV channels have been found to regulate the proliferation, apoptosis, angiogenesis, migration, and invasion of tumor cells^16^. In particular, TRPV l might serve as a therapeutic target even for the most aggressive type of breast cancer. Our results showed that tramadol could act as an agonist of TRPV1, as reported previously^17^. Although additional investigation of mechanisms is needed in relation to ER, PR, and TRPV1, tramadol can be considered to act specifically in breast cancer with ER or PR positive status and regulate TRPV1. Overall, our results suggest that tramadol may have receptor-specific antitumor effects via ER, PR and TRPV1.

Tramadol was associated with upregulating the serotoninergic system and activating the immune system by enhancing lymphocyte proliferation and NK cell activity^18^. In addition, serotonin and noradrenaline have been demonstrated to act a pivotal role in regulating the immune response; some pharmacological agents including tramadol that modulate serotonergic tone can also affect immune function^19,20^. NK cells have been known for forefront defensing against tumor cells and playing the main component of the immune system to monitor tumors, modified cells, some viral, fungal, or bacterial infections^21^. Because these roles of NK cells are to establish natural immunity and protection against tumors, NK cell activity is correlated with the life span of tumor-suppressed patients^22^. Accordingly, in breast cancer, NK cells could be effective for evaluating the prognosis of treatment^23,24^. Indeed, we found that NK cell activity was maintained in the tramadol-treated groups but decreased in the morphine group compared to control group, demonstrating a potentially greater anti-tumor immune response.

Among proinflammatory cytokines, TNF-α has a multifunctional role in regulating processes such as coagulation, metabolism, apoptosis, inflammation, and tumor growth or invasion, along with vascular functions^25^. It is also a major part of the innate inflammatory response as it promotes the expression of cytokines and chemokines, and the adhesion, extravasation, attraction, and activation of leucocytes at the site of infection^26^. IL-6 is a pro-inflammatory cytokine released by various cells in the tumor microenvironment, including cancer cells, and thus plays an important role in the expansion and differentiation of tumor cells^27,28^. Local and systemic overexpression of IL-6 has been reported in several cancer types, including breast cancer^29^. Moreover, increased IL-6 levels in the serum are generally associated with poor prognosis and low survival rates in patients with breast cancer^30^. In our findings, both TNF-α and IL-6 serum levels were reduced in the tramadol-treated xenograft mice compared with those in control mice. Accordingly, tramadol may have a positive effect on the immune system by maintaining NK cell function and suppressing proinflammatory cytokine production in patients with breast cancer.

Both in vitro and in vivo results similarly indicated that tramadol may have down-regulating actions on estrogen and progesterone receptors. Moreover, the additional anti-tumor mechanism of tramadol was confirmed in vivo by preserving NK cell activity to maintain immune function and decrease the production of pro-inflammatory cytokines such as TNF-α and IL-6. However, the mechanism related to the 5-HT_2B_ receptor has not yet been confirmed in vivo, and tramadol’s action on TRPV1 was not as clear as it was in the in vitro results. In addition, the expression of ER and PR was also significantly lower in vitro in the tramadol-treated group compared to the control group, but not in vivo statistically. The reason for these difference between in vitro and in vivo findings may be that the in vitro tramadol concentration was determined arbitrarily, whereas the in vivo tramadol concentration was determined based on the actual clinical dose.

This study has several limitations. Serial measurement of proinflammatory cytokine levels including NK cell activity over the course of tumor growth would have allowed more insights into the action of tramadol in breast cancer and offered more valuable data to our manuscript. Additionally, the precise mechanisms by which the anti-inflammatory effect of tramadol correlates directly with the immune system or reducing tumor burden needs to be identified. Given that this study was conducted in small laboratory animals, prospective human research is needed to establish the definite anti-tumor effect of tramadol in breast cancer surgery. Further research is also needed to determine the oncological mechanisms of other non-narcotic analgesics, to enable their active and appropriate use for multimodal analgesic strategies in patients with cancer.

In conclusion, the clinical dose of tramadol (1.5–3.0 mg kg^−1^) was found to attenuate the growth of breast tumors in xenograft mice. This anti-tumor effect was related to the downregulation of ERα, PRα&β, and TRPV1 expression, maintenance of immune function, especially by preserving NK cell activity, and reduced production of proinflammatory cytokines such as TNF-α and IL-6. Therefore, tramadol may have a potential effect of enhancing postoperative outcomes in patients undergoing surgery for breast cancer according to this in vivo experiment following previous our in vitro study.

## Methods

### Cell preparation and culture

We purchased MCF-7 human breast cancer cells from American Type Culture Collection (Manassas, VA, USA). And then, it was cultured in Roswell Park Memorial Institute (RPMI)-1640 medium (Hyclone, Logan, UT, USA) with l-glutamine, 10% heat-inactivated fetal bovine serum, 100 µg/mL streptomycin, and 100 IU/mL penicillin, at 37 °C in a humidified atmosphere with 5% CO_2_.

### Establishment of MCF-7 Luc cells for in vivo imaging

MCF-7 cells in complete RPMI medium were seeded in a 24-well plate at 2 × 10^4^ cells/well and incubated for 15–20 h. The medium was then changed with 500 µL of fresh complete medium containing hexadimethrine bromide (polybrene) at a final concentration of 5 µg/mL. RediFect Red-Fluc-Puro lentiviral particles (PerkinElmer, Waltham MA, USA), dissolved on ice, were supplemented with cells at a multiplicity of infection of 10. After incubating the cells with viral particles for 24 h, they were further incubated with 500 µL of fresh pre-warmed complete culture medium for 24 h. Successfully transduced cells were selected on culture plates with fresh complete culture medium with puromycin (2 µg/mL) for 3 days. MCF7-Luc cells were confirmed by assaying luciferase expression. Transduction efficiency was also determined using the Spectrum (PerkinElmer) optical in vivo imaging system (IVIS).

### Establishment of the xenograft model and sample collection

#### Ethics

All procedures related with the in vivo experiments and animal care were approved by the Committee for the Care and Use of Laboratory Animals at Yonsei University College of Medicine (no. 2017-0268) and were conducted according to the guidelines of the Care and Use of Laboratory Animals published by the US National Institutes of Health. We complied with the ARRIVE guideline 2.0 for this reporting of animal experiment conducted in the present study.

#### Animals

Female athymic nude mice (BALB/c, 6 weeks old) were obtained from the central laboratory of SLC Inc. (Hamamatsu, Shizuoka, Japan). All mice were remained in sterile cages under laminar airflow hoods in a local specific pathogen-free experimental animal facility under temperature (approximately 25 °C) and relative humidity (approximately 60%) with a 12-h light/dark cycle. We permitted free access to the tap water and sufficient chow for mice.

#### Establishment of tumors

After a 1-week acclimatization period, xenograft tumor models were established by inoculating each nude mouse with a 0.1-mL suspension of MCF7-Luc cells (5 × 10^7^ cells/mL) into the mammary fat pad.

#### Experimental groups

The xenografted mice were randomly divided into four different groups with 5 mice per group: control group (saline only), tramadol group 1 (1.5 mg kg^−1^ day^−1^ tramadol, Sigma-Aldrich, St. Louis, MO, USA), tramadol group 2 (3 mg kg^−1^ day^−1^ tramadol), and morphine group (0.5 mg kg^−1^ day^−1^ morphine; No. Seoul-1632, 1 mg/mL; Allen Care). Tramadol and morphine were freshly thawed in sterile saline prior to administration.

In addition, 5 female athymic nude mice were prepared as s sham for murine natural killer (NK) cell activity and enzyme-linked immunosorbent assay (ELISA).

#### Anesthesia and drug administration

The dose of study agents was selected to be relevant to general clinical practice, and all agents were administered through an osmotic pump (ALZET model 1004, DURECT Corp., Cupertino, CA, USA) for 28 days. The anesthesia was started with volatile agent of isoflurane 2.5–3% and maintained properly with monitoring relied on the lack of a pedal withdrawal reflex, slow but constant breathing, and the absence of response to surgical stimuli. After anesthesia was confirmed, the osmotic pump was subcutaneously implanted between the scapula of mice.

### Measurement of tumor growth

To gain a bioluminescence signal, mice bearing MCF7-Luc tumors were treated with d-luciferin (150 mg/kg, potassium salt, PerkinElmer) intraperitoneally, and placed in a light-tight mouse imaging chamber under anesthesia of 2.5–3% isoflurane. A photographic (grayscale) reference image was obtained 10 min after d-luciferin administration, and bioluminescence images were captured immediately thereafter. These images were acquired using the charge-coupled device camera cooled to − 90 °C with Spectrum IVIS (Caliper Life Sciences, Alameda, CA, USA). Regions of interest (ROIs) were marked out in the abdominal space of mice, and total photons in all areas were counted. The signal intensities of each defined ROI were calculated as the photon count rate per unit body area per unit solid angle subtended by the detector (photons s^−1^ cm^−2^ per steradian). Bioluminescence imaging signals were then recorded, and Live Imaging 4.5.5 software was used to analyze tumor growth, regression, and metastases to distant sites. The size of tumors was measured using a ruler and the tumors were weighed using a scale.

### Western blot analysis

Tissues resected from 4-week-old tumors grown in nude mice were washed with cold phosphate-buffered saline, minced, and dissolved in cell lysis buffer (Cell Signaling Technologies, Danvers, MA, USA) with protease inhibitors (10 µg/mL each of aprotinin, bestatin, l-leucine, and pepstatin A) lysed in a solution of 150 mM NaCl, 10 mM Tris, 1 mM EDTA, 1 mM benzene sulfonyl fluoride, and 1% NP-40. The total protein concentration was examined using Quick Start Bradford reagent (Bio-Rad, Hercules, CA, USA). Whole-cell extracts (50 µg) were separated on 10–15% sodium dodecyl sulphate–polyacrylamide gel electrophoresis gels and moved to the Immobilon P-transfer membranes (Millipore, Billerica, MA, USA). These membranes were then incubated with primary antibodies, which were determined using a horseradish peroxidase-conjugated IgG antibody (Cell Signaling Technologies). Bands were detected by West Glow FEMTO Chemiluminescent Substrate (BIOMAX, Seoul, South Korea). The primary antibodies targeted estrogen receptor (ER) α (1:1000, Cell Signaling Technologies), progesterone receptor PR α or β (1:1000, Cell Signaling Technologies), transient receptor potential vanilloid (TRPV)-1 (1:1000, Novus Biologicals, Centennial, CO, USA), and glyceraldehyde-3-phosphate dehydrogenase (GAPDH; 1:2000, Cell Signaling Technologies) as a loading control.

### Murine NK cell activity

Four weeks after MCF-7 cell injection, 100 µL of whole blood samples were collected in an anticoagulant (heparin, > 50 µL/mL of blood) and incubated with 30 µL of activator at 37 °C in a humidified atmosphere with 5% CO_2_ for 20–24 h. After incubation, the samples were centrifuged at 1000×*g* for 15 min. The supernatants (plasma) were then transferred to a new tube. NK cell activity in mouse blood was measured using a murine natural killer cell activity kit (NKMAX, Seoul, South Korea) as per the instructions of manufacturer.

### ELISA

Four weeks after MCF-7 cell injection, the serum levels of mouse interleukin (IL)-1β (mouse IL-1β ELISA Kit), tumor necrosis factor (TNF)-α (MHSTA50, R&D systems), interferon (IFN)-γ (Mouse IFN-gamma ELISA MAX Deluxe, Bio legend), and IL-6 (mouse IL-6 ELISA kit, Invitrogen) were measured using ELISA as per the respective instructions of manufacturer.

### Statistical analysis

Each in vitro assay was conducted in triplicate and was repeated at least three times. All data from in vivo experiments were stated as the mean (± standard deviation) and were adjusted with one-way analyses of variance with Bonferroni’s post hoc multiple comparison analysis. P-values < 0.05 were considered statistically significant. Statistical analyses were conducted using SAS version 9.4 (SAS Institute Inc., Cary, NC, USA).

## Supplementary Information

Supplementary Figure S1.

## Data Availability

The datasets used and/or analyzed during the current study are available from the corresponding author upon reasonable request.
