# Supplementary material for: Identification for antitumor effects of tramadol in a xenograft mouse model using orthotopic breast cancer cells
Source: Sci Rep. 2021 Nov 11;11:22113. doi: 10.1038/s41598-021-01701-9 (PMC8586351; doi:10.1038/s41598-021-01701-9)
Supplement: Supplementary file 1 — Supplementary Figure S1. [file 41598_2021_1701_MOESM1_ESM.docx]

**Supplementary Figure 1. The results of western blot analysis with the molecular size**


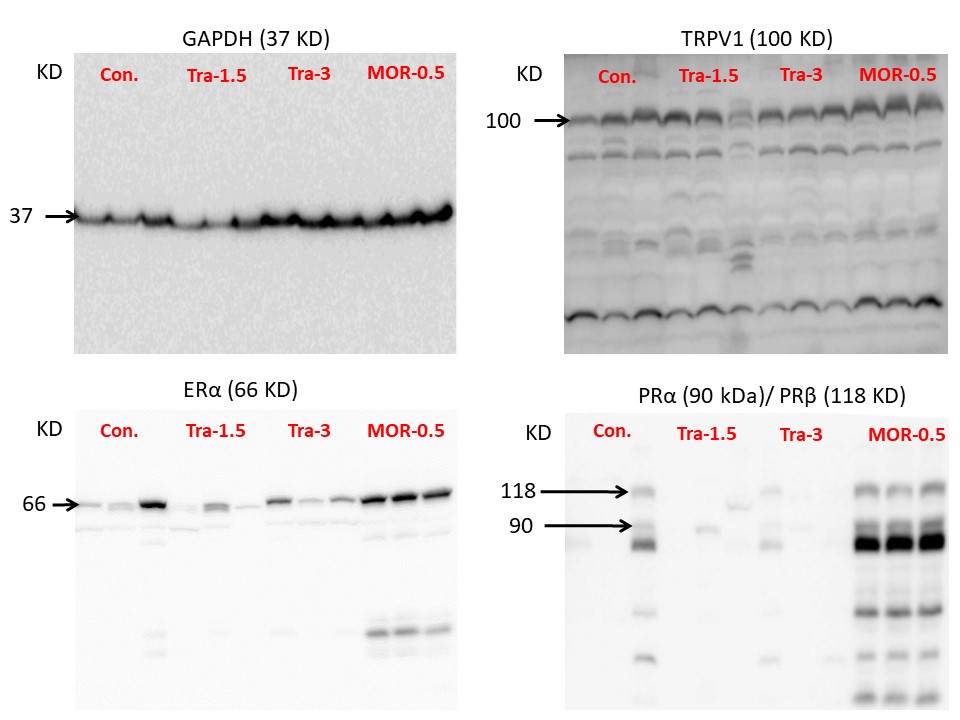


MCF, Michigan cancer foundation; GAPDH, glyceraldehyde-3-phosphate dehydrogenase; TRPV1, transient receptor potential vanilloid-1; ER, estrogen receptor; PR, progesterone receptor; Con., control group; Tra-1.5, tramadol 1.5 mg·kg^-1^·day^-1^; Tra-3, tramadol 3 mg·kg^-1^·day^-1^; MOR-0.5, morphine 0.5 mg·kg^-1^·day^-1^; KD, kilodalton
